# The Family *Borreliaceae* in Australia: Diversity, Tick Vectors, Wildlife Hosts and the Lyme Disease Controversy

**DOI:** 10.3390/microorganisms14102340

**Published:** 2026-10-14

**Authors:** Giusto Trevisan, Serena Bonin

**Affiliations:** DSM—Department of Medical Sciences, University of Trieste, 34149 Trieste, Italy; sbonin@units.it

**Keywords:** *Borreliaceae*, *Borrelia*, tick-borne diseases, Australia, wildlife hosts, Lyme borreliosis, relapsing fever, One Health

## Abstract

The family *Borreliaceae* comprises the genera *Cristispira* and *Borrelia*, the latter including the subgenera *Borrelia* and *Borreliella*. Australia hosts a unique assemblage of wildlife and tick species that may support the circulation of indigenous *Borrelia* lineages. A narrative review of the available literature was conducted to summarize current knowledge of the taxonomy, diversity, ecology, tick associations, vertebrate hosts, and potential public health significance of *Borreliaceae* detected in Australia. The diversity of Australian *Borrelia* is currently represented by members of the relapsing fever (RF) and Echidna-Reptile (REP) groups. These include *Borrelia queenslandica*, *Borrelia* sp. HB, *Candidatus Borrelia tachyglossi*, *Ca. B. undatumii*, and *Ca. B. rubricentralis*, which have been detected in association with native wildlife and tick species. However, vector competence has not yet been demonstrated for most tick-associated Borrelia lineages identified in Australia. To date, no indigenous member of the *Borrelia burgdorferi* sensu lato complex has been convincingly detected in Australian ticks, wildlife, or humans. Nevertheless, persistent symptom complexes following tick exposure have been reported and are currently grouped under the umbrella term Debilitating Symptom Complexes Attributed to Ticks (DSCATT). Current evidence indicates that Australian *Borreliaceae* fauna is dominated by indigenous relapsing fever and reptile-associated lineages rather than Lyme borreliosis agents. Further genomic, ecological, reservoir-host, and vector-competence studies are needed to clarify their transmission cycles, evolutionary relationships, zoonotic potential, and their significance within a One Health framework.

## 1. Introduction

The family *Borreliaceae* comprises the genera *Cristispira* and *Borrelia*, a group of spirochaetes characterized by a distinctive helical morphology, periplasmic flagella, and complex genomes consisting of a linear chromosome and multiple linear and circular plasmids [1,2]. Members of this family occupy a wide range of ecological niches and are associated with diverse vertebrate and invertebrate hosts worldwide. Climate change, environmental modification, and increasing human–wildlife interactions influencing the distribution of ticks and tick-borne microorganisms, including *Borrelia* species [3]. Within the family, the genus *Borrelia* comprises three major phylogenetic groups: the Lyme borreliosis (LB) group, the relapsing fever (RF) group, and the Echidna-Reptile (REP) group [4]. Although the taxonomic status of *Borreliella* remains debated, recent classifications generally recognize *Borreliella* as a subgenus encompassing the Lyme borreliosis agents, whereas relapsing fever and reptile-associated species remain within the subgenus *Borrelia* [5,6].

Australia harbors a distinctive fauna of vertebrates and tick community, with approximately 70 recognized tick species reported across the continent [7]. Within the broader Australasian region, tick diversity comprises 125 tick species, belonging to the families Ixodidae (102 species) and Argasidae (23 species) [8]. Australia’s long geographic isolation has resulted in unique hosts and tick communities that differ markedly from those found in Lyme borreliosis-endemic regions [8,9]. Recent molecular investigations have identified several indigenous *Borrelia* lineages associated with native wildlife, particularly echidnas, reptiles, monitor lizards, and rodents. These lineages belong predominantly to the RF and REP clades [10,11,12,13].

In contrast, despite decades of investigation, there is no convincing evidence supporting the existence of endemic Lyme borreliosis caused by members of the *Borrelia (Borreliella) burgdorferi* sensu lato (Bbsl) complex in Australia [14,15,16]. Nevertheless, persistent symptom complexes occurring after tick exposure continue to fuel debate regarding Lyme-like illnesses in Australia and are currently grouped under the term Debilitating Symptom Complexes Attributed to Ticks (DSCATT) [17].

This review provides an updated overview of *Borreliaceae* detected in Australia, with a focus on taxonomy, phylogenetic diversity, tick associations, wildlife hosts, and potential implications for animal and human health. Particular attention is given to indigenous Australian *Borrelia* species, their ecological transmission cycles, and the ongoing discussion surrounding Lyme borreliosis and DSCATT.

## 2. Material and Methods

This narrative review was based on a structured literature search of the PubMed, Scopus, and Web of Science databases. The search was conducted in March 2026 and included publications from database inception through February 2026. Search terms comprised combinations of keywords related to *Borrelia*, Australia, borreliosis, tick-borne pathogens, tick vectors, ecology, epidemiology, and surveillance. Only articles published in English were considered. Peer-reviewed original research articles, reviews, government surveillance reports, and publicly available health agency publications relevant to indigenous *Borrelia* species and their associated tick vectors in Australia were included. Conference abstracts lacking sufficient methodological details, duplicate publications, studies focused exclusively on imported cases, and articles for which full text was unavailable were excluded. When multiple publications reported overlapping datasets, the most comprehensive or recent study was preferentially included to avoid duplication of evidence. The reference lists of eligible publications were also screened to identify additional relevant studies. Although this review was not conducted as a systematic review and did not follow PRISMA guidelines, efforts were made to enhance transparency and reproducibility using a predefined search strategy and explicit eligibility criteria.

## 3. Taxonomy and Phylogeny of the Family *Borreliaceae*

### 3.1. The Family Borreliaceae

The family *Borreliaceae* belongs to the phylum *Spirochaetota* and comprises helical, motile bacteria characterized by periplasmic flagella located between the outer membrane and the protoplasmic cylinder. These host-associated microorganisms exhibit a distinctive spiral morphology and possess complex genomes consisting of a linear chromosome together with multiple linear and circular plasmids, a genomic architecture that distinguishes them from most other bacterial taxa [1,2].

The family currently includes two genera, *Cristispira* and *Borrelia*. Phylogenomic analyses based on conserved molecular signatures and comparative genomic studies support their classification within *Borreliaceae*, which represents a distinct lineage within the order *Spirochaetales* [18].

Members of *Borreliaceae* occupy diverse ecological niches and maintain complex associations with a wide range of vertebrate and invertebrate hosts. Several members are transmitted by arthropod vectors, particularly hard and soft ticks, and are of considerable veterinary and medical importance. Environmental changes, including climate change and anthropogenic habitat modification, are increasingly recognized as factors influencing tick distribution and the circulation of tick-borne microorganisms, including *Borrelia* species [19].

#### The Genus Cristispira

The genus *Cristispira* is the second recognized genus within the family *Borreliaceae*. These large uncultivable spirochaetes occur primarily within the crystalline style of marine and freshwater bivalve mollusks, where they are generally regarded as non-pathogenic symbionts or commensals [1,18,20,21]. They are distinguished from other spirochaetes by the presence of a characteristic ridge-like structure, known as the *crista*, formed by an exceptionally large bundle of periplasmic flagella located between the outer membrane and the protoplasmic cylinder [22,23]. Because *Cristispira* species have not been successfully cultivated in vitro, their phylogenetic placement relies largely on 16S rRNA analyses, which consistently identify them as a sister lineage to the genus *Borrelia* within the family *Borreliaceae* [18,21]. Members of the genus have been reported mainly in oysters, particularly *Cristispira* *pectinis*, which inhabits the crystalline style of *Crassostrea virginica* [24,25].

In Australia, the principal oyster species are *Crassostrea gigas* and *Saccostrea glomerata*. However, despite extensive investigations of oyster-associated microbiota, neither *Cristispira* nor any other member of the family *Borreliaceae* has been reported to date [26].

### 3.2. Current Taxonomic Classification

The taxonomy of *Borreliaceae* has undergone substantial revision over the past decade. Traditionally, all medically relevant species were classified within the genus *Borrelia*. However, comparative genomic analyses subsequently proposed dividing the genus into two distinct genera, *Borrelia* and *Borreliella*, based on conserved sequence insertions and deletions, conserved signature proteins, and genomic similarity metrics such as average nucleotide identity and average amino acid identity [5].

The proposed reclassification generated considerable debate. While *Borreliella* was validly proposed as a separate genus encompassing the Lyme borreliosis group, its acceptance remains controversial, and both taxonomic frameworks continue to be used in the literature. Alternative phylogenetic interpretations have proposed retaining a single genus, *Borrelia*, while recognizing *Borreliella* as a subgenus [4,6].

For this review, the major phylogenetic lineages within *Borreliaceae* are referred to as:

- the Lyme borreliosis (LB) group;
- the Relapsing Fever (RF) group; and
- the Echidna-Reptile (REP) group [4,6].

These groups differ in their evolutionary history, host associations, vector relationships, and pathogenic potential. Lyme borreliosis agents are primarily associated with *Ixodes* ticks and mammalian reservoirs, whereas relapsing fever borreliae are typically associated with soft ticks of the family Argasidae, although several species are transmitted by hard ticks. REP borreliae constitute a distinct lineage predominantly associated with reptiles, echidnas, monitor lizards, and the tick that parasitize these hosts [27,28].

### 3.3. Relevance of Borreliaceae in Australia

Australia harbors a highly distinctive fauna shaped by its prolonged geographic isolation and supports approximately 70 recognized tick species, most oof which belong to the families Ixodidae and Argasidae [7,8]. The country’s wildlife includes numerous endemic vertebrates’ species that may serve as hosts for indigenous *Borrelia* lineages, creating ecological systems that differ markedly from those described in North America, Europe, and Asia [8,29].

Recent molecular studies have shown that the Australian *Borrelia* fauna is dominated by members of the relapsing fever and REP clades. Indigenous taxa identified to date include *Borrelia queenslandica*, *Borrelia* sp. HB, *Candidatus Borrelia tachyglossi*, *Ca. Borrelia undatumii*, and *Ca. Borrelia rubricentralis*. These organisms have been detected in association with native rodents, echidnas, monitor lizards, and the ticks that parasitize them [11,12,13,30,31,32].

In contrast, despite extensive surveillance using culture-based methods, polymerase chain reaction assays, and next-generation sequencing technologies, no convincing evidence supports the existence of endemic Lyme borreliosis caused by members of the Bbsl complex in Australia [14,15,16]. Nevertheless, persistent symptom complexes following tick exposure continue to attract scientific and public attention and are currently classified under the term Debilitating Symptom Complexes Attributed to Ticks (DSCATT) [17].

The following sections review the diversity of Australian ticks and their associated *Borrelia* species, with particular emphasis on relapsing fever borreliae, reptile-associated lineages, and their potential implications for animal and human health.

## 4. Tick Diversity in Australia

### 4.1. Australian Tick Fauna

Ticks are obligate hematophagous ectoparasites of vertebrates and represent some of the most important vectors of pathogens affecting wildlife, domestic animals, and humans. Australia harbors a remarkably diverse tick fauna, with around 70 recognized species distributed across a broad range of habitats, from tropical and temperate forests to arid inland regions. These species belong predominantly to the families Ixodidae (hard ticks) and Argasidae (soft ticks), while representatives of other tick lineages are absent or extremely rare in the Australian fauna [8].

Hard ticks (Ixodidae) are characterized by the presence of a dorsal scutum and prolonged feeding periods that may last several days. They include members of the genera *Ixodes*, *Amblyomma*, *Bothriocroton*, *Haemaphysalis*, and *Rhipicephalus*. In contrast, soft ticks (Argasidae) lack a dorsal scutum and feed more rapidly; they are represented mainly by species of the genera *Ornithodoros* and *Argas* [8,33].

Australian ticks parasitize a wide range of wildlife hosts, including marsupials, monotremes, birds, reptiles, and bats. Large-scale surveys have shown that marsupials, birds, and reptiles are among the most frequently infested hosts, reflecting the unique evolutionary history and biodiversity of the Australian continent [34]. The distribution and abundance of ticks are influenced by multiple ecological factors, including temperature, humidity, vegetation structure, host density, and host movements. Environmental change, habitat modification, and climate change are increasingly recognized as drivers of shifts in tick distribution and may contribute to the emergence of novel tick-borne pathogens and vector–host associations in Australia [34,35].

### 4.2. Tick Species of Medical and Veterinary Importance

Although most Australian tick species primarily parasitize wildlife, several species can infest humans and domestic animals. More than 20 tick species have been reported biting humans, with *Ixodes holocyclus* representing the most medically important species because of its ability to cause tick paralysis through the injection of potent neurotoxins [33,35,36]. Other human-biting species include *Ixodes cornuatus*, *Ixodes tasmani*, *Ixodes australiensis*, *Haemaphysalis bancrofti*, *Ornithodoros gurneyi*, and *Ornithodoros capensis* [33,35,37]. Beyond their role as vectors, Australian ticks may directly affect human and animal health through paralysis, hypersensitivity reactions, local inflammatory lesions, and the induction of alpha-gal syndrome, a tick-associated allergy to mammalian meat products that has been linked primarily to *Ixodes holocyclus* and, more recently, to *Ixodes australiensis* [36,38,39]. Recent investigations have shown that Australian ticks harbor a wide range of microorganisms, including bacteria, protozoa, and viruses, many of which remain poorly characterized. These findings highlight the importance of continued surveillance of Australian tick populations and their associated microbiota within a One Health framework [10,34,40].

### 4.3. Tick Identification and Surveillance Approaches

Accurate tick identification is fundamental for understanding the epidemiology of tick-associated microorganisms and assessing potential vector competence. Traditionally, Australian ticks have been identified on the basis of morphological characteristics using optical microscopy, stereomicroscopy, and dichotomous taxonomic keys. However, morphological similarity among species, particularly within genera such as *Haemaphysalis*, can complicate species-level identification [33,34]. To overcome these limitations, molecular methods have become increasingly important. Polymerase chain reaction (PCR) assays targeting mitochondrial and nuclear markers are widely used for species confirmation and pathogen detection. DNA sequencing approaches, including Sanger sequencing and next-generation sequencing (NGS), have considerably expanded our understanding of Australian tick biodiversity and facilitated the discovery of previously unrecognized microorganisms and novel *Borrelia* lineages [10,33,40].

Metagenomic and metatranscriptomic approaches are increasingly being employed to characterize the microbial communities associated with Australian ticks. These technologies provide powerful tools for detecting emerging pathogens, investigating host-vector-pathogen interactions, and identifying novel microorganisms that may be relevant to both wildlife and public health [10,12,40].

### 4.4. Australian Ticks Associated with Borrelia Detection

Several Australian tick species have been found to harbor indigenous *Borrelia* species or have been repeatedly associated with Borrelia DNA in molecular studies. These include soft ticks such as *Ornithodoros gurneyi* and hard ticks belonging to the genera *Haemaphysalis*, *Bothriocroton*, *Amblyomma*, and *Ixodes*. Molecular investigations have demonstrated associations between these ticks and members of both the relapsing fever and Echidna-Reptile groups of *Borrelia* [11,12,13,31,32,41]. In contrast, despite extensive surveillance efforts involving culture-based methods, PCR assays, and NGS technologies, no Australian tick species has been conclusively shown to harbor endemic members of the *Borrelia burgdorferi* sensu lato complex responsible for Lyme borreliosis in the Northern Hemisphere [14,15,42]. Consequently, understanding tick diversity, host associations, and vector competence remains essential for clarifying the ecology of indigenous Australian *Borrelia* species and assessing their potential significance for animal and human health.

## 5. Relapsing Fever Borreliae in Australia

### 5.1. Soft Tick-Borne Relapsing Fever

Relapsing fever borreliae are traditionally associated with soft ticks of the family Argasidae, particularly species of the genus *Ornithodoros*. In well-characterized transmission systems, these spirochaetes circulate in enzootic cycles involving vertebrate hosts and competent tick vectors, with persistence facilitated by transstadial and, in some species, transovarial transmission. While soft tick-borne relapsing fever (STBRF) is well documented in Africa, Asia, Europe, and the Americas, evidence for indigenous STBRF borreliae in Australia remains scarce [28,43].

#### 5.1.1. *Borrelia queenslandica*

The first indigenous Australian RF borrelia was described in 1962 as *Borrelia queenslandica*, following its isolation from the long-haired rat (*Rattus villosissimus*) in Queensland [30]. Experimental studies demonstrated that this spirochaete could infect mice, whereas guinea pigs appeared resistant. DNA of *Borrelia queenslandica* has been detected in association with the kangaroo soft tick *Ornithodoros gurneyi* and is thought to occur in native rodents, including *Rattus villosissimus* [41].

No evidence of human pathogenicity has been reported to date [30]. Subsequent molecular investigations confirmed the presence of *Borrelia*-related DNA in Australian ticks associated with the same ecological setting, supporting the continued circulation of this lineage among native wildlife and associated tick populations in the study area [44]. However, compared with RF borreliae described in other continents, the ecology and host range of *B. queenslandica* remain poorly understood [30,41].

#### 5.1.2. *Ornithodoros gurneyi*

The soft tick *Ornithodoros gurneyi* is the tick species most strongly associated with *B. queenslandica*. This argasid tick inhabits arid and semi-arid regions of Australia, where it parasitizes a variety of vertebrate hosts, including rodents, kangaroos, and reptiles [44,45,46]. However, vector competence and transmission capacity have not yet been experimentally demonstrated. Although primarily associated with wildlife, *O. gurneyi* occasionally bites humans [47]. Reported reactions include local inflammation and systemic manifestations such as headache, visual disturbances, vomiting, and malaise [33,47]. Nevertheless, human infection with *B. queenslandica* has never been reported [30,41].

#### 5.1.3. Other Australian Argasidae

Several additional argasid ticks occur in Australia, including *Ornithodoros capensis*, *Argas robertsi*, *Argas persicus*, *Argas (Carios) dewae* [33,44,48,49,50,51]. These species parasitize seabirds, poultry, bats, and other wildlife and occasionally bite humans [33,49,50,51]. To date, molecular surveys have failed to identify RF borreliae in most Australian Argasidae. Studies of *O. capensis* collected from seabird colonies did not detect *Borrelia* DNA [45]. Similarly, no indigenous borreliae have been identified in *A. robertsi* and *A. dewae*, despite investigations of their associated microbial communities [45,46,49,52,53]. Consequently, the association between *B. queenslandica* and *O. gurneyi* represents the best-supported candidate soft tick-borne relapsing fever system currently recognized, although vector competence remains to be demonstrated [30,41].

### 5.2. Hard Tick-Borne Relapsing Fever

Hard tick-borne relapsing fever (HTBRF) borreliae differ from classical relapsing fever borreliae by their association with ixodid ticks rather than argasid ticks. In Australia, recent molecular and genomic studies have identified a previously uncharacterized hard tick-associated lineage provisionally designated *Borrelia* sp. HB [12,40].

#### 5.2.1. *Borrelia* sp. HB

The identification of *Borrelia* sp. HB represents one of the most significant recent advances in the understanding Australian borreliae diversity. This organism was initially identified through metatranscriptomic analyses of ticks and wildlife from New South Wales and was subsequently characterized through targeted molecular investigations [12,40]. Phylogenetic analyses placed *Borrelia* sp. HB within the relapsing fever clade while demonstrating its distinction from currently recognized species [12]. Phylogenetically, the organism is closely related to several cervid-associated and hard tick-associated relapsing fever borreliae reported from Asia [12,54].

Despite considerable efforts, *Borrelia* sp. HB has not yet been successfully cultivated in vitro. Short-lived spirochaete-like structures were observed during culture attempts, but sustained growth could not be achieved [12]. The inability to culture this organism currently limits detailed characterization of its biology, ecology, and pathogenic potential [12].

#### 5.2.2. Relationship with *Borrelia theileri*

Interest in Australian hard tick-borne relapsing fever borreliae predates the discovery of *Borrelia* sp. HB. Earlier reports described spirochaetes isolated from Australian cattle that showed morphological similarities to *Borrelia theileri*, a relapsing fever species transmitted by hard ticks and widely distributed in Africa, Asia, and other regions [54,55,56,57]. Although these observations suggested the presence of *B. theileri*-like organisms in Australia, molecular confirmation was not available at the time [55]. More recent genomic analyses indicate that *Borrelia* sp. HB represents a distinct lineage and is more closely related to Asian hard tick-associated RF borreliae than to Lyme borreliosis agents [12,54].

#### 5.2.3. *Haemaphysalis bancrofti* as a Putative Vector

*Haemaphysalis bancrofti* is the tick species most frequently associated with the detection of *Borrelia* sp. HB in Australia [12,58]. However, vector competence has not yet been experimentally demonstrated, and further studies are required to clarify its role in the maintenance and transmission of this organism. *Haemaphysalis bancrofti* parasitizes a broad range of vertebrate hosts, including eastern gray kangaroos (*Macropus giganteus*), red-necked wallabies (*Notamacropus rufogriseus*), livestock, companion animals, and occasionally humans [8,58]. Although *H. bancrofti* exhibits a relatively broad host range, the prevalence of *Borrelia* sp. HB in sampled ticks appears to be relatively low [12]. Furthermore, extensive screening of wildlife hosts has revealed very limited evidence of infection, suggesting that the enzootic cycle of this organism may be highly restricted or involve currently unidentified vertebrate hosts that contribute to the maintenance of the organism in nature [12,40]. At present, no human infections attributable to *Borrelia* sp. HB have been documented [12]. Nevertheless, its discovery highlights the previously underestimated diversity of indigenous Australian RF borreliae and emphasizes the need for further investigations into host associations, vector competence, zoonotic potential, and successful cultivation of Australian *Borrelia* species [12,40]. The major hard tick-borne relapsing fever borreliae, their associated tick species, host associations, and geographic distribution are summarized in Table 1.

### 5.3. Louse-Borne Relapsing Fever

Louse-borne relapsing fever (LBRF) is caused by *Borrelia recurrentis* and is transmitted by the human body louse (*Pediculus humanus corporis*). Unlike other relapsing fever borreliae, which are primarily maintained through tick-associated enzootic cycles, *B. recurrentis* is restricted to humans and does not require an animal reservoir [28,67]. Historically, LBRF has been responsible for large epidemics and remains endemic in parts of East Africa, particularly Ethiopia and neighboring countries in the Horn of Africa [67,68]. Sporadic imported cases have also been reported in Europe, mainly among migrants and travelers arriving from endemic regions [67].

To date, no locally acquired cases of louse-borne relapsing fever have been reported in Australia, and there is no evidence that *B. recurrentis* circulates within the country [68]. Consequently, LBRF currently has limited relevance to the epidemiology of indigenous Australian borreliae but remains an important consideration in the differential diagnosis of febrile illness in travelers returning from endemic regions [67,68].

**Figure 1:**
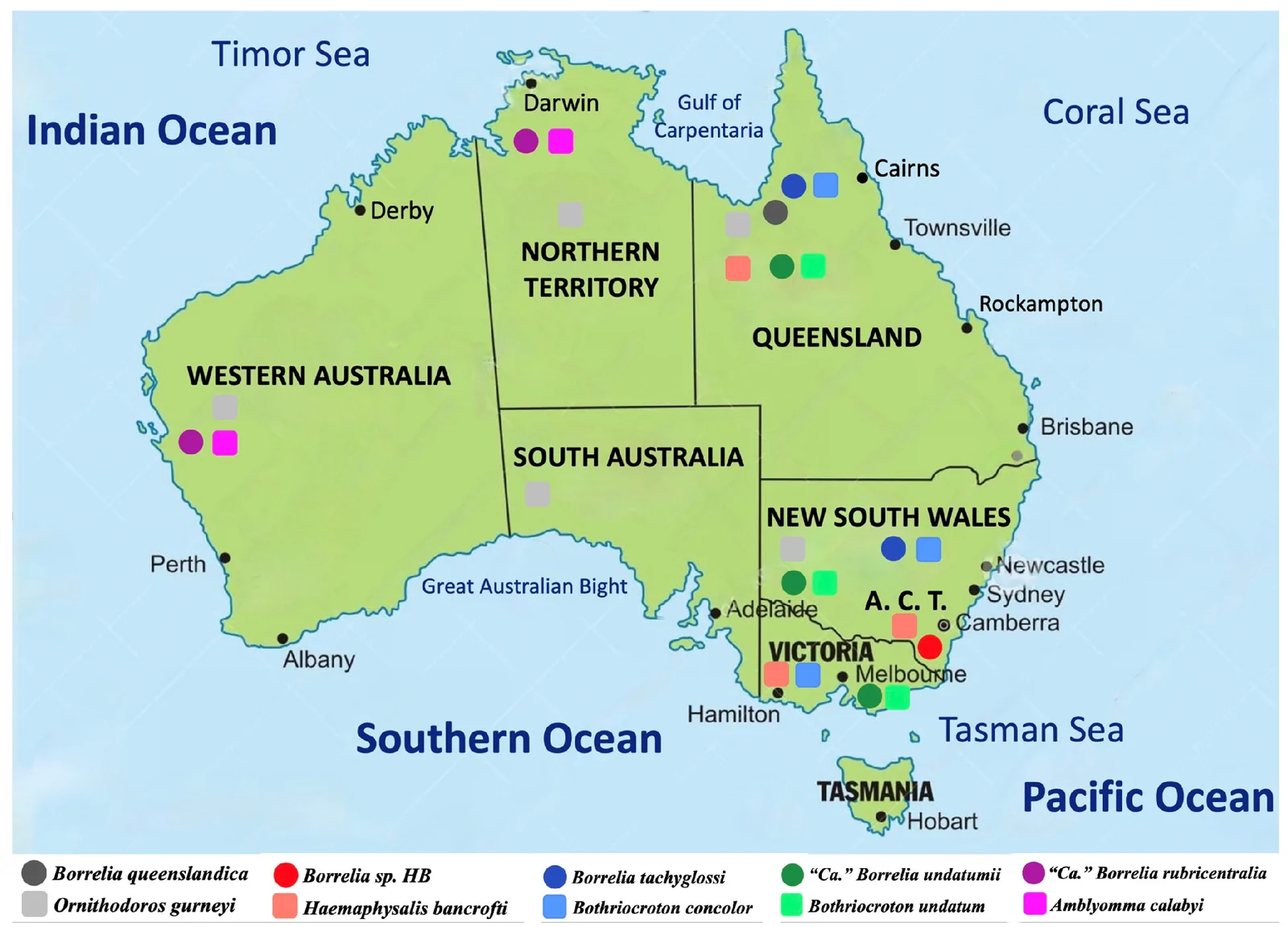
Geographic distribution of indigenous *Borrelia* species identified in Australia and their tick species from which they have been detected or with which they have been associated in published studies. State labels indicate the Australian states where detections were reported and do not represent precise collection localities. *Borrelia* species are shown as colored circles, whereas associated tick species are indicated by lighter-colored squares. The figure does not imply confirmed vector competence or transmission cycles unless demonstrated experimentally.

The geographic distribution of the indigenous Australian *Borrelia* species identified to date, including relapsing fever and reptile-associated lineages, together with their putative tick vectors, is shown in Figure 1.

## 6. REP Group Borreliae

### 6.1. Evolutionary Position of REP Group Borreliae

Reptile-associated borreliae constitute a distinct evolutionary lineage within the genus *Borrelia*. Initially considered an unusual group of limited biological significance, REP borreliae are now recognized as a distinct clade within the genus *Borrelia.* Phylogenetic analyses based on multiple genetic markers indicate that REP borreliae form a lineage distinct from both Lyme borreliosis and relapsing fever borreliae [27,69]. The first member of this lineage, *Borrelia turcica*, was isolated from *Hyalomma aegyptium* ticks collected from *Testudo graeca* in Turkey and subsequently characterized using culture, molecular, and phylogenetic approaches [70]. Since then, several additional REP borreliae have been identified in reptiles, birds, echidnas, and their associated ticks from Europe [71,72], Asia, Africa [73,74], Australia, and South America, revealing a broader host range and geographic distribution than originally recognized [11,69,75]. Current evidence suggests that REP borreliae comprise several host-associated evolutionary lineages, including turtle-, bird-, echidna-, and monitor lizard-associated groups [11]. Their ecology and pathogenic potential remain incompletely understood, although no confirmed human infections have been documented to date [11,27].

### 6.2. Australian REP Borreliae

Australia represents one of the most important hotspot of REP borrelial diversity currently recognized. Most indigenous *Borrelia* species identified on the continent belong to this clade and have been detected in association with native wildlife, particularly echidnas and monitor lizards [11,13,31]. The first Australian REP borrelia to be characterized was *Candidatus Borrelia tachyglossi*, identified in *Bothriocroton concolor* ticks parasitizing the short-beaked echidna (*Tachyglossus aculeatus*) in Queensland [13,31]. Subsequent genomic analyses demonstrated that *Ca. B. tachyglossi* possesses a relapsing fever-like genomic architecture while remaining phylogenetically embedded within the REP lineage [76]. Several additional Australian REP species have subsequently been described. *Candidatus Borrelia undatumii* was detected in *Bothriocroton undatum* ticks collected from *Varanus varius* in eastern Australia [32]. Similarly, *Candidatus Borrelia rubricentralis* was identified in *Amblyomma calabyi* and *Amblyomma fimbriatum* parasitizing *Varanus giganteus* in arid and semi-arid regions of Australia [11]. Comparative genomic studies have revealed remarkable diversity among these monitor lizard-associated borreliae and support the existence of a distinct Australasian lineage within the REP clade [11].

### 6.3. Vertebrate Hosts and Tick Associations

Unlike Lyme borreliae, which are predominantly maintained by rodent–bird cycles, REP borreliae display a strong association with reptiles and other evolutionarily distinctive vertebrate hosts [27,69]. In Australia, REP borreliae have most frequently been detected in ticks of the genera *Bothriocroton* and *Amblyomma* [11,31,32]. *Bothriocroton concolor* is closely associated with echidnas and is the tick species from which *Ca. B. tachyglossi* has most consistently been detected [13,31]. Likewise, *Bothriocroton undatum* has been reported in association with the detection of *Ca. B. undatumii* in *Varanus varius* [32]. Monitor lizards appear to be particularly important hosts for REP borreliae. Species such as *Varanus giganteus*, *Varanus varius*, and *Varanus salvator* have been associated with genetically distinct *Borrelia* lineages distributed throughout Australia and Southeast Asia [11,77,78]. The ecological specialization observed among REP borreliae suggests a long evolutionary history of adaptation between these spirochaetes, their associated tick species, and reptilian hosts [11]. Although some associated ticks occasionally parasitize other vertebrates, there is currently no evidence supporting transmission of REP borreliae to humans [11,32].

### 6.4. Biogeography of the REP Group

The REP clade is distributed across a broad geographic area extending from southeastern Europe and the Middle East to Australia, Southeast Asia, and South America [69]. Outside Australia, REP borreliae have been identified in *Amblyomma geoemydae* and *Amblyomma helvolum* ticks associated with reptiles in Japan and Thailand [69,79]. In Indonesia and Thailand, *Borrelia salvadorii* has been detected in *Amblyomma varanense* ticks parasitizing the Asian water monitor (*Varanus salvator*) [77,78]. The geographic range of the REP lineage was further expanded by the isolation of *Borrelia mahuryensis* from passerine-associated *Amblyomma* ticks in French Guiana [75]. This finding suggests that REP borreliae are not restricted to reptiles and may circulate in more complex ecological networks involving birds and potentially other vertebrate hosts [75].

Phylogenetic analyses indicate that monitor lizard-associated borreliae from Australia and Southeast Asia form a well-supported monophyletic group within the broader REP clade [11]. The discovery of multiple novel REP species in Australia over the past decade highlights the continent as a major center of evolution and diversification for reptile-associated *Borrelia* [11,31,32]. The currently recognized REP borreliae, their reported tick associations, vertebrate hosts, and geographic distribution are summarized in Table 2.

## 7. Lyme Borreliosis and the Australian Debate

### 7.1. Lyme Borreliosis Worldwide

Lyme borreliosis is the most common tick-borne bacterial disease in the Northern Hemisphere and is caused by members of the Bbsl complex. Transmission involves ticks of the genus *Ixodes* and a variety of vertebrate reservoir hosts, particularly rodents, birds, and small mammals [27,63,81]. More than 25 species have been assigned to the LB group, several of which are recognized human pathogens [6,63]. In North America, Lyme borreliosis is transmitted primarily by *Ixodes scapularis* and *Ixodes pacificus*, with *Borrelia burgdorferi* sensu stricto representing the predominant etiological agent [82,83]. In Europe, *Ixodes ricinus* serves as the principal vector and transmits several pathogenic species, including *B. afzelii*, *B. garinii*, *B. bavariensis*, and *B. burgdorferi* sensu stricto [63,84]. In Asia, *Ixodes persulcatus* is the major vector and contributes to the circulation of *B. afzelii* and *B. garinii* [63]. Lyme borreliosis has also been reported sporadically in Central and South America [81]. The typical clinical manifestation is erythema migrans, which develops in approximately 70–80% of infected individuals. If left untreated, infection may affect the nervous system, joints, skin, heart, and other organ systems [63]. The global distribution of Lyme borreliosis closely follows that of competent *Ixodes* vectors and their vertebrate reservoirs [81,85].

### 7.2. Evidence from Australia

Although several species of *Ixodes* ticks are present in Australia, including *Ixodes holocyclus*, *I. cornuatus*, *I. tasmani*, and *I. australiensis*, current evidence does not support the existence of an endemic transmission cycle involving members of the *Borrelia burgdorferi* sensu lato (Bbsl) complex [7,35,37,86]. Numerous studies have investigated Australian ticks, wildlife, domestic animals, and human samples using culture-based methods, PCR-based assays, serology, and NGS technologies [14,42]. Furthermore, a combination of serological tests was employed to investigate sentinel dogs from regions with high incidence of *Ixodes holocyclus* ticks, with all samples testing negative for Bbsl [42]. Despite differences in sampling strategies, diagnostic targets and analytical sensitivity among studies, no indigenous member of the Bbsl complex has been conclusively identified in Australia [14,15,42].

Serological studies have occasionally reported positive or equivocal reactions. For example, indirect immunofluorescence testing performed in Queensland during the late 1980s yielded positive results in a subset of patients; however, these findings were not confirmed using more specific methodologies, such as Western blot, and were therefore considered inconclusive [87]. Subsequent evaluations highlighted important limitations of Lyme borreliosis (LB) serology in Australia, including the possibility of cross-reactivity with other microorganisms and reduced specificity when used outside endemic settings [88]. Some reports have described patients presenting with symptoms resembling Lyme borreliosis following tick exposure in Australia, including erythematous skin lesions and musculoskeletal complaints [89,90]. However, these observations have not been supported by definitive microbiological evidence demonstrating infection with members of the Bbsl complex and therefore should not be interpreted as evidence of an endemic Australian Lyme borreliosis cycle [14,15,90].

Imported Lyme borreliosis should be distinguished from the question of endemic transmission. Laboratory-confirmed cases have been documented in Australian residents who acquired infection while traveling or living in endemic regions of Europe or North America and were diagnosed after returning to Australia [15,91]. These cases demonstrate that Lyme borreliosis can occur in Australian residents; however, they do not constitute evidence of local transmission within Australia.

### 7.3. Debilitating Symptom Complexes Attributed to Ticks (DSCATT)

In response to reports of persistent symptoms following tick exposure, and in absence of evidence supporting endemic Lyme borreliosis, Australian authorities adopted the descriptive term Debilitating Symptom Complexes Attributed to Ticks (DSCATT). DSCATT is not a single disease entity or etiological diagnosis but rather an umbrella term encompassing a heterogeneous symptom complexes reported following tick exposure [14,17]. Patients classified as DSCATT frequently report prolonged fatigue, musculoskeletal pain, neurological complaints, cognitive symptoms, sleep disturbances, and reduced quality of life [17]. Although these manifestations may overlap with symptoms reported in Lyme borreliosis, available evidence does not support a common etiology [14,17]. A case-series analysis of Australian patients with DSCATT highlighted the heterogeneous nature of the condition and emphasized the absence of microbiological evidence linking these symptoms to Bbsl infection [17]. Consequently, the underlying mechanisms of DSCATT remain poorly understood and warrant further investigation through multidisciplinary clinical and laboratory studies [17].

### 7.4. Why Has Lyme Borreliosis Not Become Established in Australia?

Several hypotheses have been proposed to explain the apparent absence of endemic Lyme borreliosis in Australia; however, direct evidence supporting any single explanation remains limited. Proposed explanations include Australia’s long geographic isolation [9], differences in host community composition, [9,85] and distinctive tick ecology and host-seeking behavior compared with those observed in Northern Hemisphere ecosystems [9,85,92,93]. Taken together, the available evidence indicates that the indigenous Australian *Borrelia* fauna is dominated by relapsing fever and REP lineages rather than by members of the Lyme borreliosis group [11,12,15,42].

## 8. Discussion

### 8.1. Wildlife, Ticks and Indigenous Borrelia Diversity

Australia hosts more than 8000 described vertebrate species and a highly diverse tick fauna adapted to a wide range of ecological niches [29,33]. Recent molecular investigations have demonstrated that wildlife-associated ticks harbor a broad range of microorganisms, including several previously undescribed *Borrelia* lineages [10,12,34,40]. Based on the currently available evidence, most indigenous Australian *Borrelia* taxa belong to either the RF or REP clades and have been detected in association with native rodents, echidnas, monitor lizards, bats, and other wildlife hosts [11,12,30,31,32]. However, the vertebrate reservoirs, transmission dynamics, and ecological significance of many of these organisms remain poorly understood [12,34].

Interpretation of molecular detection studies requires caution. Many reports are based on broad-range PCR assays targeting short fragments of genes such as 16S rRNA or *flaB*, approaches that may have limited phylogenetic resolution and are potentially susceptible to contamination or misclassification when sequence similarity among taxa is high. Likewise, metagenomic analyses depend on the completeness and accuracy of available reference databases. Furthermore, detection of *Borrelia* DNA in engorged ticks does not necessarily indicate persistent colonization or vector competence, as detected nucleic acids may originate from the vertebrate blood meal rather than from an established infection of the tick. These limitations should be taken into account when interpreting reports of novel *Borrelia* lineages and proposed tick-host associations.

### 8.2. Climate Change and Environmental Modification

Climate change and environmental modification may influence the distribution of Australian ticks and their vertebrate hosts, potentially altering opportunities for pathogen transmission [94,95,96]. However, evidence linking these environmental changes specifically to the distribution, emergence, or transmission dynamics of Australian *Borrelia* species remains limited. Future ecological studies integrating tick surveillance, wildlife monitoring, and molecular pathogen detection will be essential to determine whether climate-driven changes affect the distribution and epidemiology of indigenous Australian borreliae [34,94,95,96].

### 8.3. Knowledge Gaps and Research Priorities

Despite significant advances in molecular detection technologies, several important questions remain unresolved. Many Australian *Borrelia* species have not yet been successfully cultivated, limiting investigations of their pathogenicity, host range, and evolutionary relationships [12,97].

Future studies should prioritize:

- the isolation and cultivation of indigenous Australian *Borrelia* species;
- the identification of vertebrate reservoir hosts;
- the evaluation of vector competence in Australian tick species;
- the assessment of zoonotic potential through experimental and genomic approaches; and
- long-term surveillance integrating wildlife, veterinary, and human health data [12,34,35,40].

Although members of the genus *Cristispira* are of evolutionary interest within the family *Borreliaceae,* information regarding their occurrence in Australia remains extremely limited and their relevance to tick-borne disease ecology remains unknown [1,18,26].

Integrating ecological, microbiological, veterinary, and medical expertise within a One Health framework will be essential for improving our understanding of the ecological and public health significance of Australian borreliae [34,98].

## 9. Conclusions

The Australian *Borreliaceae* fauna is dominated by indigenous relapsing fever and REP-group lineages that are distinct from the classical Lyme borreliosis agents circulating in the Northern Hemisphere [11,12,30,31]. Current evidence supports the presence of species such as *Borrelia queenslandica*, *Borrelia* sp. HB, *Candidatus Borrelia tachyglossi*, *Ca. Borrelia undatumii*, and *Ca. Borrelia rubricentralis* in wildlife and in tick species from which their DNA has been detected [11,12,30,31,32]. In contrast, no endemic transmission cycle involving members of the *Borrelia burgdorferi* sensu lato complex has been convincingly demonstrated in Australia [14,15,17,42].

Further studies integrating genomics, ecology, vector competence research, and One Health surveillance will be essential to clarify the evolutionary history, ecological significance and potential public health relevance of Australian *Borrelia* species [12,34,98].

## Figures and Tables

**Table 1:** Hard Tick-Borne Relapsing Fever Worldwide and in Australia.

Borrelia	Associated Hard Ticks	Geographical Areas	Main Hosts/Reservoirs	References
*B. lonestari*	*Amblyomma americanum*	Eastern and central USA	White-tailed deer (*Odocoileus virginianus*), birds	[59]
*B. miyamotoi*	*Ixodes persulcatus*, *I. ricinus*, *I. scapularis*	Eurasia, North-America, limited reports from Africa	Rodents are major reservoir hosts, also reported in birds and camels	[60,61,62]
*B. theileri*	Mainly *Rhipicephalus (Boophilus) microplus*, also reported from *R. turanicus*, *R. annulatus*, *Haemaphysalis longicornis* and *H. flava*	Africa, Asia and Americas	Primarily cattle, occasional reports in goats, dogs and rodents	[63,64,65,66] [55]
*Borrelia* sp. HB	*Haemaphysalis bancrofti*	Australia (New South Wales)	Wallabies and kangaroos; Tick occasionally bites humans	[12]

**Table 2:** REP-Group Borrelia species reported worldwide and in Australia.

*Borrelia* Species	Host Lineage	Vertebrate Hosts	Associated Tick Species	Geographic Distribution	References
*B. turcica*	Turtles	*Testudo graeca*	*Hyalomma aegyptium*	South-East Europe, Asia	[70]
*B. tachyglossi*	Monotremes	Short-beaked echidna (*Tachyglossus aculeatus*)	*Bothriocroton concolor*, *Ixodes holocyclus*	Australia (Queensland, New South Wales)	[31]
*B. mahuryensis*	Birds	Passerine birds	*Amblyomma longirostre*, *A. geayi*	French Guiana	[75]
*Borrelia* REP spp.	Lizard, turtles	Monitor lizards (*Varanus exanthematicus*)	*Amblyomma geoemydae*	Thailand, Japan	[69,79,80]
*B salvatorii*	Monitor Lizards	*Varanus salvator*	*Amblyomma varanense*	Indonesia (West Java), Thailand	[77,78]
*“Ca.” Borrelia undatumii*	Monitor lizards	*Varanus varius*	*Bothriocroton undatum*, *Amblyomma varanense*	Australia (Queensland, New South Wales, Victoria)	[32]
*“Ca.” Borrelia rubricentralis*	Monitor lizards	*Varanus giganteus*	*Amblyomma calabyi* and *A. fimbriatum*	Australia (Western Australia, Northern Territory)	[11]

## Data Availability

No new data were generated or analyzed in this study. Data sharing is not applicable to this article.

## Abbreviations

The following abbreviations are used in this manuscript:

Bbsl	*Borrelia burgdorferi* sensu lato
CLL	Chronic lymphocytic leukemia
DSCATT	Debilitating Symptom Complexes Attributed to Ticks
FlaB	Flagellin B gene
HTBRF	Hard tick-borne relapsing fever
LB	Lyme borreliosis
NGS	Next generation sequencing
PCR	Polymerase chain reaction
PRISMA	Preferred Reporting Items for Systematic reviews and Meta-Analyses
REP	Echidna-Reptile group
RF	Relapsing fever
16S rRNA	16S ribosomal RNA gene
STBRF	Soft tick-borne relapsing fever
Sp.	Species
